# Low-Dose Naltrexone Co-Treatment in the Prevention of Opioid-Induced Hyperalgesia

**DOI:** 10.7759/cureus.17667

**Published:** 2021-09-02

**Authors:** Gurneet Shaheed, Anthony P Manjooran, Akshay J Reddy, Neel Nawathey, Samuel Habib, Hetal Brahmbhatt

**Affiliations:** 1 Department of Chemistry and Biochemistry, University of California, Los Angeles, Los Angeles, USA; 2 Genetics, Arizona State University, Phoenix, USA; 3 Opthalmology, California Northstate University College of Medicine, Elk Grove, USA; 4 Health Sciences, California Northstate University, Rancho Cordova, USA; 5 Health Sciences, Santa Clara University, Santa Clara, USA; 6 Psychiatry, Mercy General Hospital, Sacramento, USA

**Keywords:** opiod abuse, naltrexone, opioid-induced hyperalgesia, pain management, tolerance

## Abstract

Opioid-induced hyperalgesia (OIH) is characterized by a heightened sensitivity to pain that occurs in patients following opioid use. Prescription of opioids is currently the standard form of pain management for both neuropathic and nociceptive pain, due to the relief that patients typically report following their use. Opioids, which aim to provide analgesic effects, can paradoxically cause increasing degrees of pain among the users. The increased nociception can be either due to the underlying pain for which the opioid was initially prescribed, or other unrelated pain. As a result, those who are initially prescribed opioids for chronic pain relief may instead be left with no overall relief, and experience additional algesia. While OIH can be treated through the reduction of opioid use, antagonistic treatment can also be utilized. In an attempt to reduce OIH in patients, low doses of the opioid antagonist naltrexone can be given concurrently. This review will analyze the current role and effectiveness of the use of naltrexone in managing OIH in opioid users as described in clinical and non-clinical studies. Additionally, it seeks to characterize the underlying mechanisms that enable opioid antagonist naltrexone to reduce OIH while still allowing opioids to act as an analgesic. The authors find that OIH is a prevalent condition, and in order to effectively combat it, clinicians and patients can benefit from an extended study on how naltrexone can be utilized as a treatment alongside opioids prescribed for pain management.

## Introduction and background

With over 153 million written prescriptions a year, opioids are the predominant treatment for chronic pain and postoperative pain in the United States. As opioids are considered the most effective analgesics, they are often used for moderate to severe nociceptive and neuropathic pain that is not cured by weaker medications [1]. Paradoxically, opioid-induced hyperalgesia (OIH) is a long-term outcome of the use of prescription opioid analgesics, where patients experience heightened nociception following the use of opioids. Patients with OIH experience heightened sensitivity to innocuous or noxious stimuli due to their opioid tolerance [1]. This often prompts clinicians to increase the dosage of opioids, which in turn increases pain in patients. This condition is associated with a dysfunction of the endogenous opioid system [2].

Currently, there is no definitive treatment for patients experiencing OIH; however, there have been numerous trials to attempt to understand the effect of the concurrent use of opioid antagonists to prevent or treat OIH. One treatment that has shown significant efficacy in trials to treat OIH is the use of naltrexone, an opioid antagonist typically used to treat substance abuse. While naltrexone was not originally designed to treat OIH, it has been shown to improve symptoms in low doses through its mechanism as a pure opioid receptor antagonist [1-2]. However, very high doses of naltrexone can lead to side effects such as insomnia and hallucinations, and hence the administration of the opioid antagonist should be conducted by adhering to the guidelines set by healthcare professionals [2]. In order to examine and understand the effects of naltrexone as a co-treatment with opioids in an objective manner, the cold pressor test (CPT) can be utilized as an objective measure of pain [2-3]. This review will further discuss methods of characterizing treatment for OIH, as well as evaluate the results of clinical studies that have explored the effects of naltrexone as a treatment for OIH.

## Review

OIH is characterized as a paradoxical phenomenon whereby treatment of both acute and chronic pain with opioids results in increased nociceptive sensitivity. As such, patients will experience heightened levels of pain to certain stimuli from even a single morphine administration [4]. Although this consequence of opioid use has been known for many decades, efforts to study and mitigate OIH have been inadequate until recently. Fortunately, ongoing research in the field of pain management has provided various approaches to reduce or even potentially eliminate the short and long-lasting implications of OIH. As indicated in Table 1, the most potent mitigating effects on OIH are achieved when opioids are administered concurrently with low doses of opioid antagonists, most commonly naltrexone, or naloxone in some cases [1-26]. Naltrexone is traditionally administered to treat withdrawal symptoms associated with opioid use disorders and functions as a competitive antagonist of the μ-opioid receptor (MOR), and to lesser extents the κ-opioid receptor and δ-opioid receptor [5]. Similarly, naloxone functions as a high-affinity competitive antagonist of the MOR and the σ-opioid receptor. Due to the subjective nature of analyzing differences between baseline nociceptive sensitivity and OIH-induced nociceptive sensitivity, it is critical to analyze the various conformations and dynamic mechanisms regulating the G-protein-coupled MOR in response to ultra-low doses of naltrexone following, during, and prior to opioid use.

**Table 1:** Classifications and effective dosages of opioid agonists and corresponding antagonists

Author (year)	Opioid agonist	Effective dosage of the agonist	Opioid antagonist	Effective dosage of the antagonist
Apfel et al. (1995) [14]	N/A	N/A	Naloxone	15.0 mg/kg
Augusto et al. (2019) [15]	N/A	N/A	Naltrexone	10.0 mg/kg
Baamonde et al. (2005) [16]	Morphine	1.0 μg/kg	Naloxone	2.0 mg/kg
Campillo et al. (2011) [17]	Remifentanil	80 μg/kg	Naloxone	1.0 mg/kg
Corder et al. (2017) [13]	Morphine	10 mg/kg	Naltrexone	0.9 mg/kg
Crain and Shen (2001) [7]	Morphine	1.0 μg/kg	Naltrexone	0.1 ng/kg
Crain and Shen (2008) [12]	Morphine	1.0 μg/kg	Naltrexone	0.1 ng/kg
Cruciani et al. (2003) [8]	Oxycodone	5.0 mg every 6 hours	Naltrexone	1.0 μg x 2 per day
Harris et al. (2004) [4]	Morphine	10 mg/kg	Naloxone	2.5 mg/kg
Jackson et al. (2021) [2]	Morphine	N/A	Naltrexone	0.1 mg/kg
Juni et al. (2006) [19]	Morphine	0.1 μg/kg	Naltrexone	100 pg/kg
Largent-Milnes et al. (2008) [6]	Oxycodone	10 mg/kg	Naltrexone	1.0 μg/kg
Le Roy et al. (2011) [20]	Fentanyl	50 ng/kg	Naltrexone	1.0 mg/kg
Oaks et al. (2018) [3]	Morphine	4.5 mg per day	Naltrexone	0.1 mg x 2 per day
Pineda-Farias et al. (2017) [10]	Morphine	N/A	Naltrexone	0.5 ng/kg
Podolsky et al. (2013) [22]	Morphine	N/A	Naloxone	1 mg/kg
Terashvili et al. (2007) [5]	Morphine	N/A	Naltrexone	2.3 pg/kg
Van Dorp et al. (2009) [11]	Morphine	0.29 mg/kg	Naltrexone	10 mg/kg
Walwyn et al. (2016) [23]	Morphine	19 μg/kg	Naltrexone	10 mg/kg
Wang et al. (2005) [9]	Morphine	2.5 μg every 48 hours	Naloxone	1.0 mg/kg
Wang et al. (2008) [24]	Morphine	10 mg/kg	Naloxone	1.0 μg/kg
Waxman (2009) [25]	Fentanyl	10 mg/kg	Naltrexone	0.05 mg/kg
Whitehouse (1985) [26]	Morphine	N/A	Naltrexone	10 mg/kg

Recent research has demonstrated that in the presence of abundant exogenous opioids, there occurs a shift in (MOR)-G-protein coupling from the default inhibitory subtype G_i/o_ to the hyperalgesic excitatory G_s_. The data referenced in Table 1 suggests that the most commonly utilized opioid agonist for co-treatment with naltrexone was morphine [1-26]. When endogenous opioids or very minuscule concentrations of opioids bind the MOR, inhibitory G_i_ or G_o_ proteins are recruited to the G-protein-coupled receptors (GPCRs) and activated. Subsequent signaling results in diminished levels of cyclic adenosine monophosphate and hyperpolarization due to heightened potassium efflux and reduced calcium influx, leading to reduced pain transmission [6]. Current clinically recommended doses of opioids have been shown to cause OIH by paradoxically shifting recruitment of inhibitory G_i_ to excitatory G_s_ proteins, further amplifying pain propagation and the development of OIH. In fact, a controlled study analyzing nociceptive neurons in mice demonstrated that morphine and similar opioids can elicit MOR G-protein-mediated hyperalgesic effects at doses 1,000-fold less than the dose necessary to elicit an analgesic effect, as measured by tail-flick assay [7-8]. These findings are further supported by the work of Cruciani and Pasternak who preferentially downregulated the G_s_ regulatory protein by intrathecal injection of antisense oligonucleotides in mice and similarly observed reductions in low-dose morphine-induced hyperalgesia [8]. As such, co-treatment of both neuropathic and nociceptive pain with standard doses of morphine in conjunction with ultra-low-dose naltrexone rapidly reverses, and in most cases prevents, the undesired MOR G_i/o_ to G_s_ transition along with reductions in analgesic tolerance, withdrawal symptoms, and dependence induced by opioid usage [9]. In addition, as per receptor theory, the addition of an opioid antagonist to an opioid agonist presumably requires increasing the amount of prescribed agonist to achieve the same analgesic effect. However, because naltrexone functions to reduce unwanted conformational changes in the G-protein of MORs, a lower dose of the opioid agonist, than what is traditionally prescribed, is needed to achieve the same or even more potent analgesic effects.

In addition to mitigating unwanted changes or internalization of the MOR and other similar opioid receptors, several studies have reported significant increases in the analgesic potency of opioids when co-treated or pre-treated with ultra-low-dose (10 ng/kg) naltrexone [10-11]. Moderate doses of morphine (0.1-3 mg/kg) in conjunction with low-dose naltrexone in mice progressively increased the magnitude and duration of analgesia beyond treatment with morphine alone. Even ultra-low dose (1 μg/kg) morphine plus extreme-low-dose naltrexone (0.1 ng/kg) has shown a significant enhancement of opioid antinociceptive potency [7]. More recent studies in rats have clinically demonstrated that implantation of 30-mg naltrexone pellets significantly elevate naltrexone plasma levels and sustain pharmacologically functional levels of naltrexone such that a 50-fold rightward shift of the morphine analgesia dose-response curve is observed a full eight days later [11]. These findings show great promise toward reducing the clinically recommended dosage of opioids to treat neuropathic and nociceptive pain by both minimizing the dosage of prescribed opioids and maximizing the time between dose administrations. Additionally, because naltrexone, when administered in ultra-low doses, has a high binding affinity for the specific conformation of high-efficacy excitatory (hyperalgesic) G_s_-coupled opioid receptors, no side effects or adverse events are likely to occur with co-treatment of opioid agonists [12].

Despite all the progress that has been made in the efforts to understand OIH so far, further research is still required in order to develop a more permanent treatment for those who suffer from this condition and are far beyond the threshold for naltrexone co-treatment. Perhaps other medications that break down the production of beta-lipoproteins, such as the metal-chelator ethylenediaminetetraacetic acid (EDTA), could be used in stabilizing and analyzing nociceptive and μ-opioid receptors for more novel treatment options [27]. Additionally, more research should be conducted to determine the side effects of naltrexone so that patients can make a more informed decision before their physician provides them with treatment options. In the future, there could potentially be investigations into the human proteome utilizing a western blot analysis that could help us understand more about the G-protein receptors that are involved in the pathology of this disease [28-29]. Hopefully, additional treatment options could be devised to benefit those who have developed pervasive OIH and cannot be co-treated with naltrexone due to the difficult-to-reverse end-stage progression of this disease. Similarly, perhaps in the future, machine learning could be used to help diagnose patients with this rare condition so that more clinical data would be readily available to foster further scientific understanding of OIH [30].

Due to the exacerbation of the opioid epidemic, it is becoming increasingly crucial to develop preventative measures around opioid abuse and addiction at a healthcare level. It is necessary for healthcare providers to reiterate the detrimental side effects of opioid use, including tolerance and OIH, which counteract opioid analgesia and drive dose escalation [13]. As such, naltrexone holds great potential as a preventative medicine rather than a cure to an already developed opioid dependence. In fact, when used to reverse acute morphine withdrawals in albino rats, repeated naltrexone administrations exacerbated the severity of potentiated startle and hyperalgesia [4]. In contrast, a very recent clinical study involving 55 human patients diagnosed with OIH and treated with low-dose naltrexone reported over a quadrupled level of pain tolerance as measured by the CPT and confirmed by statistical analysis (p<0.0001) [2]. These findings indicate that opioid antagonists are most effective when administered concurrently with the desired opioid agonist. Therefore, it is critical to introduce naltrexone treatment prior to or during opioid treatment to eliminate any severe symptoms of withdrawal that overcome the diminishing analgesic effects of opioids.

## Conclusions

Based on the studies mentioned above in this review, it is clear that naltrexone, naloxone, and other related opioid antagonists show great promise as a treatment for OIH due to their properties as selective substrates of the MORs when combined in low doses with opioid prescriptions. This is especially important due to the scope and enormity of the ongoing opioid epidemic in the United States. The review shows the potential of naltrexone, when administered in doses as low as 0.3 mg twice a day, as a co-treatment with opioids, in preventing OIH and reducing the dosage of opioids overall. Additionally, naltrexone co-treatment reduces the necessary dosage for analgesia when prescribing opioids, thereby helping to mitigate the risks of over-prescription and abuse. Future studies should be conducted to further understand the effect of naltrexone as a treatment for OIH when taking prescription opioids. Future studies should also aim to gain a deeper understanding of the mechanisms by which OIH arises in patients. Practitioners should give more attention to naltrexone and other opioid antagonists as a potentially viable treatment for OIH.
